# The *in vivo* function of the p53 target gene TIGAR

**DOI:** 10.1186/1753-6561-6-S3-P12

**Published:** 2012-06-01

**Authors:** Eric C Cheung, Dimitris Athineos, Rachel Ridgway, Karen Blyth, Douglas Strathdee, Owen Sansom, Karen H Vousden

**Affiliations:** 1 The Beatson Institute for Cancer Research, Glasgow, G61 1BD, UK

## 

The p53 tumour suppressor inhibits tumour development via various mechanisms such as apoptosis, inhibition of proliferation or the activation of senescence. Recently, several studies have indicated a novel role of p53 in the regulation of energy metabolism. Previously we have discovered TIGAR, a p53 target gene that acts as a fructose-2,6-bisphosphatase. TIGAR therefore can redirect glucose from the glycolytic pathway to the pentose phosphate pathway (PPP), which promotes NADPH production to generate reduced glutathione for protecting against ROS, and also ribose 5 phosphate production for nucleotide synthesis. In order to understand the function of TIGAR *in vivo*, we generated TIGAR deficient mice. We have determined a critical role of TIGAR in rapidly proliferating tissue, either for repair after damage or during tumor development.

